# Geriatric depression: prevalence and its associated factors in rural Odisha

**DOI:** 10.3389/fpubh.2023.1180446

**Published:** 2023-06-15

**Authors:** Annu Antony, Swayam Pragyan Parida, Priyamadhaba Behera, Susanta Kumar Padhy

**Affiliations:** ^1^Department of Community Medicine and Family Medicine, AIIMS Bhubaneswar, Bhubaneswar, Odisha, India; ^2^Department of Psychiatry, AIIMS Bhubaneswar, Bhubaneswar, Odisha, India

**Keywords:** geriatric, depression, prevalence, rural Odisha, elderly, cross-sectional study

## Abstract

The world's population is aging rapidly, and the epidemiological transition has led to increased mental disorders worldwide. Geriatric depression is masked by multiple comorbidities or the natural process of aging. Our study aims to estimate the prevalence of geriatric depression and find the risk factors associated with geriatric depression in rural Odisha. The study was a multistage cross-sectional study conducted in the Tangi block, district Khordha, Odisha, from August 2020 to September 2022, among 520 participants selected by probability proportional to size sampling. From the selected participants, eligible 479 older adults were interviewed using a semi-structured interview schedule, Hindi Mini Mental Scale, Geriatric Depression Scale-15, and Hamilton Depression Rating Scale. The step forward multivariable logistic regression was used to assess the associated factors of depression among older adults. Among our participants, 44.4% (213) of older adults were depressed. Substance abuse in family members [AOR: 16.7 (9.1–30.9)], history of elder abuse [AOR: 3.7 (2.1–6.7)], physical dependency [AOR: 2.2 (1.3–3.6)], and financial dependency [AOR: 2.2 (1.3–3.6)] are significant independent risk factors associated with geriatric depression. Living with children [AOR: 0.33 (0.18–0.59)] and recreational activity [AOR: 0.54 (0.34–0.85)] are significant protective factors of geriatric depression. Our study found that geriatric depression is highly prevalent in rural Odisha. Poor quality of family life and physical and financial dependency was found to be the most significant risk factor for geriatric depression.

## Introduction

The world's population has been rapidly aging for the last 50 years. The National Policy on Older Adults defines “senior citizen” or “geriatric population” as 60 years and above (1). The global geriatric population has doubled from 1990 to 2019 by 703 million (2) United Nations (UN) world geriatric population prospects estimate the global aging population to double again by 2050 and is projected to reach nearly 1.5 billion. Western Asia, including India, expects an increase in the geriatric population of ~230% (2). Being prepared to address the needs of the growing older adult population is a necessity. According to the WHO, the prevalence of geriatric depressive disorders varies from 10 to 20% in different regions (3, 4). A meta-analysis on the prevalence of depression among older adults (60 years and above) in India from 1997 to 2016 revealed that 34% of older adults in India suffer from various depressive disorders (5).

Depression is a common and severe disorder affecting our feelings and daily activities (6). The individual usually suffers from depressed mood, loss of interest, and reduced energy leading to increased fatiguability and diminished activity (7). Depressed older adults are less likely to endorse their symptoms, and they often attach them to existing physical illnesses (8). Addressing the growing burden of depressive disorders in the older adults of India is possible by increasing awareness about depressive disorders in older adults, timely diagnosis, and treatment. According to the WHO, the treatment gap (the number of people with a disease who are not in treatment) for mental illness in developing countries is as high as 75–85% (9). India's recent National Mental Health Survey (NMHS) revealed an 85% treatment gap for depressive disorders in India (10). Older adults are vulnerable to immobility and dependency, facing even more difficulty getting diagnosed and treated. Considering the facts from the metanalysis of the prevalence of geriatric depression, which states that 34% of older adults suffer from depression and that there is an 85% treatment gap for depression according to the National Mental Health Survey, we expect a vast number of undiagnosed older adults with depressive disorders (5, 10). The prevalence of depression varies in different regions and age groups (5). The risk factors in older adults also vary due to gender, sociocultural practices, and geographical areas (11). Apart from this, limitations in daily activities and social alienation can also be understood as major risk factors for depression in older adults (8, 12). Consequently, we hypothesized that some specific risk factors of older adults residing in rural areas could increase the risk of depression. Therefore, our study aims to assess the prevalence of depression to understand the burden of depressive disorders among older adults in rural Odisha and explore the risk factors of depression among them.

## Materials and methods

Our study was a community-based cross-sectional study in Tangi Block in Khordha district in Odisha State in Eastern India. The block has a total of 6 sectors and 154 villages. The field practice area of AIIMS Bhubaneswar under Tangi RHTC consists of six sectors.

**Figure 1:**
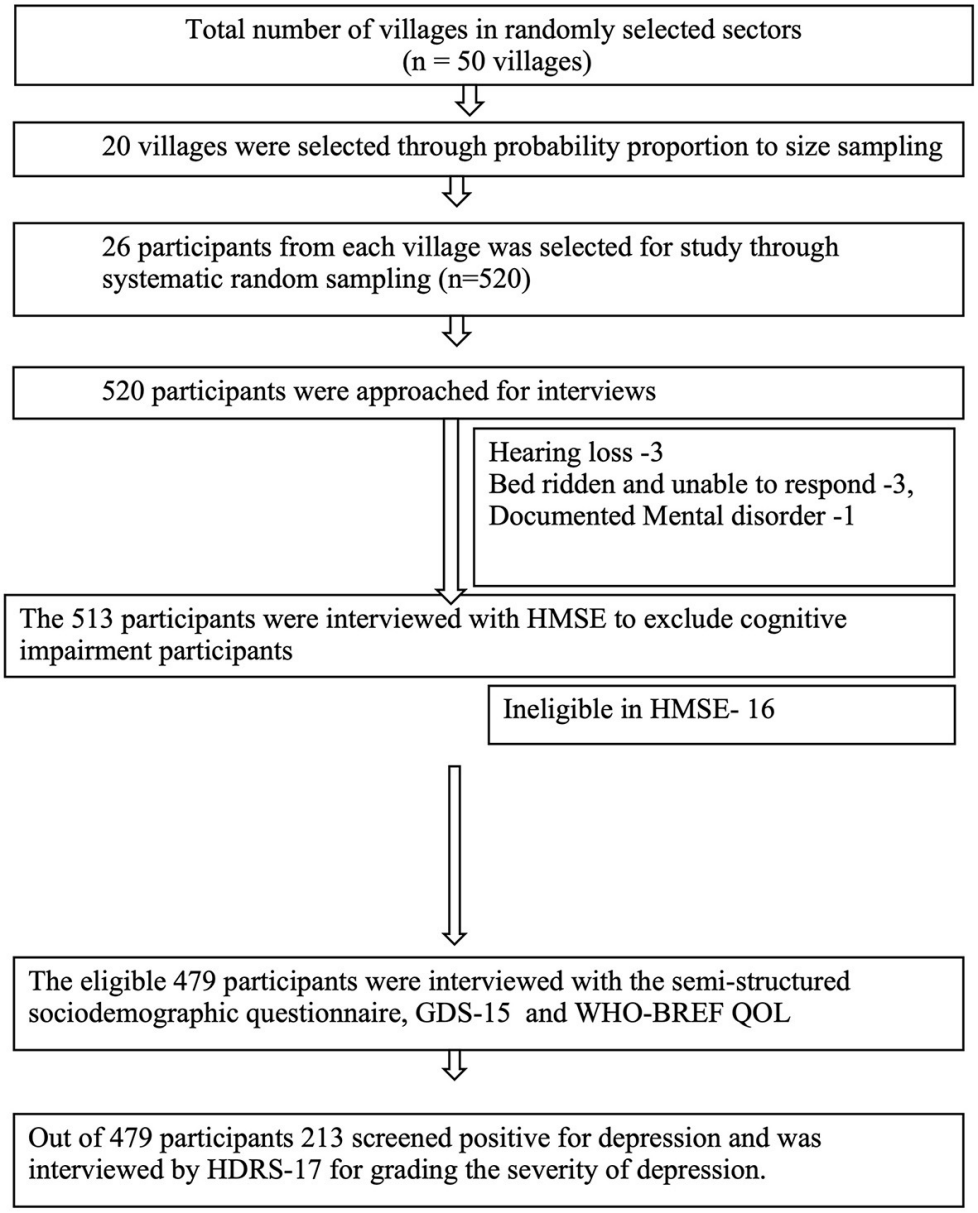
Study flow chart.

**Figure 2:**
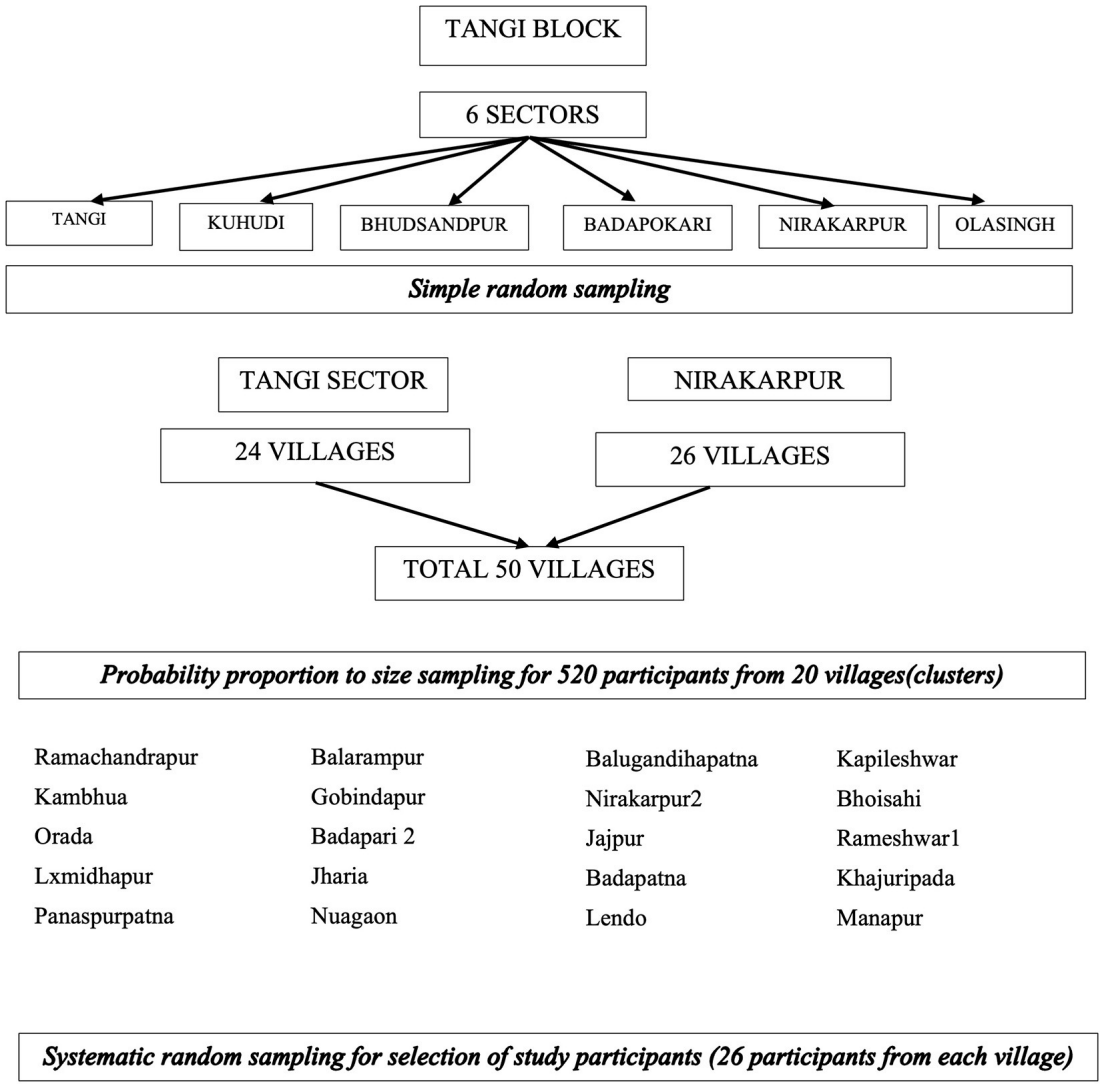
Sampling strategy.

The study population was older adults aged 60 years and above residing in TANGI BLOCK, District Khordha, during the data collection period from August 2020 to September 2022. The COVID-19 pandemic affected the study period due to the lockdowns and restrictions. Assuming a prevalence of 37.9%, a relative error of 15%, and a confidence level of 95%, the sample size was 296. Furthermore, taking a design effect of 1.5, the sample size needed was 444. After considering the exclusion as 5% and the non-response rate as 10%, the final sample estimated was 519 persons aged 60 years and above. Our sampling strategy was probability proportional to the size sampling of 20 villages. The sample per village was rounded off to 26 after dividing the sample size 519 among 20 villages (25.9 and a total sample size of 520 participants were interviewed (Figure 1). All persons aged 60 years and above residing in this area for at least the last 6 months were included in the study. Older adults with impaired cognition (assessed by Hindi Mental State Examination) and those who were unable to respond because they had hearing loss, were unable to speak, had no comprehension, or were too sick to respond were excluded, as the GDS-15 Questionnaire could not be applied to those participants. Our sampling strategy involved multistage sampling, as explained in Figure 2.

### Data collection and statistical analysis

Data were collected using one-to-one interviews at the participant's house after ensuring privacy. A single investigator who was trained in the psychiatry department of AIIMS Bhubaneswar conducted the interviews. Depression was screened using the Geriatric Depression Scale 15 (GDS-15)—Odia after ruling out older adults with impaired cognition using the Hindi Mental State Examination (HMSE). The GDS-15-Hindi was translated into Odia and validated (linguistic, face, and content validity) among older adults from a similar study area. A cut-off of 8 was taken for screening for depression as per GDS-15 in Indian settings (13, 14). The depression of participants screened positive by GDS-15 was quantified by the Hamilton depression rating scale−17 (HDRS-17). Sociodemographics and other factors associated with depression were assessed using a semi-structured questionnaire. The data were entered into Epicollect5 after data validation. The datasheet was cleaned for missing and inappropriate variables, and the final variables were coded in Microsoft Excel 365 for analysis. Statistical analysis was done using SPSS 28. Categorical variables were summarized by percentages. A logistic regression analysis was used to assess possible risk factors. All variables with univariable association < 0.25 and universal confounders such as age, gender, and education were included in the step forward multivariable logistic regression model with a probability of entry and removal of 0.05 and 0.10, respectively, to identify independent risk factors.

The risk factors were calculated as odd ratios (OR) with 95% confidence intervals (CI). The significance of differences was defined as a two-tailed *p*-value of < 0.05.

## Results

Among the sample of 520 older adults identified by our study, 497 were found to be eligible for the study. Out of the 497 participants, 21 did not give consent for participation. Thus, the non-response rate was only 3.6% for our research, and coverage of 96.4% (479) was achieved. Gender was almost equally distributed among our participants, with 55.8% (267) women and 44.2% (212) men, and 73% (350) of the participants were in the younger age group (60–74 years old). In comparison, only 7% (15) of the participants were in the older age group (> 85 years). Older adults residing in joint or three-generational families constituted 59.5% (285), and older adults living with their spouses constituted 66.2% (194). Most of them were also illiterate (41.9%, 201), unemployed (52.4%, 251), and had lower socioeconomic status (53.2%). It was also observed that 48.5% of our participants had medical insurance (Table 1).

**Table 1:** Distribution of sociodemographic variables.

**S. No**.	**Baseline characteristics**	**Category**	**Frequency**	**Percentage**
1	Gender	Women	214	55.8
		Men	283	44.2
2	Religion	Hindu	462	96.4
		Muslim	17	3.6
3	Age	Younger age group (60 to 74 years)	352	73.1
		Middle age group (75 to 84years)	105	19.8
		Older age group (>/= 85 years)	40	7.1
4	Marital status	Married and living with spouse	162	66.2
		Unmarried/divorced/Separated	317	33.8
5	Type of family	Joint family and three generational family	285	59.5
		Nuclear family	194	40.5
6	Education	Illiterate	201	41.9
		Read only	82	17.3
		Read and write	41	8.5
		Primary	59	12.3
		Middle	48	10
		High and above	48	10
7	Employment	Unemployed	251	52.4
		Unskilled	112	23.4
		Semi-skilled	9	1.8
		Skilled	40	8.4
		Semi-professional	25	5.3
		Professional	42	8.7
8	Poverty line based on annual income	APL	395	83.5
		BPL	78	16.4
9	House status	No house	59	12.3
		Kucha	111	23.1
		Mixed	125	26.1
		Pucca	184	38.4
10	SES (according to Uday Pareek's scale)	Lower class	59	53.2
		Upper Lower	111	40.9
		Lower Middle	125	5.2
		Upper middle	184	0.7
11	Insurance	No	246	51.5
		Yes	233	48.5

The proportion of studied older adults who screened positive for depression was 44.4% (213) (Table 2). Most of them, 74% (158), had mild depression, while 15% (16) had moderate depression and 2% (4) had severe depression (Table 3). According to our screening, 58% (124) of older adult men were found to be depressed compared to 33% (89) of older adult women. The prevalence of depression was highest in the middle age group (75–84 years = 50%, 48) than in both the younger (150, 42.8%) and older age group (15, 44.11%).

**Table 2:** Prevalence of depression among older adults with GDS 15.

	**Frequency**	**Percentage (CI)**
Depressed	213	**44.4% (40.02–48.92)**
Non-depressed	266	55.6% (50.92–60.04)
Total	479	100%

**Table 3:** Severity scoring with HDRS.

**HDRS**	**Frequency (N= 213)**	**Proportion (n=213)**
No depression	20	9.38%
Mild	158	74.2%
Moderate	31	14.5%
Moderately severe	0	0
Severe	4	1.9%
Total	213	

The prevalence of depression among participants living without a spouse was 77.7% (126) and among participants from nuclear families was 50% (97). Older adults residing with their children had a prevalence of only 40% (156) compared to 64% (57) among older adults living without their children. Prevalence of depression was higher in participants with adverse events in the family (129, 49.4%), substance use among family members (96, 65.7%), and elder abuse (68, 72.3%).

Prevalence of depression was lower in the older adults with physical activity (108, 39.8%), recreational activity (91, 35.5%), and social participation (63, 39.8%), and was higher in those who were physically dependent on others for self-care (6, 75%) and financially dependent older adults (173, 64.07%). There was no difference in the prevalence of depression among older adults with current comorbidities (182, 44.8%) and without comorbidities (31, 42.4%). Similarly, the prevalence of depression in older adults with non-communicable chronic diseases was 45.08% [179 and in those without non-communicable chronic diseases, it was 41.4% (15)].

Step forward multivariable logistic regression was applied, considering depression as the dependent variable and sociodemographic and other factors as the independent variable. The model was statistically significant, with 46.9% of the variability in depression among the older adults explained [R^2^ (Nagelkerke): 0.469].

We found that substance abuse in family members [AOR: 16.7 (9.1–30.9)], history of elder abuse [AOR: 3.7 (2.1–6.7)], physical dependency for self-care [AOR: 2.2 (1.3–3.6)], and financial dependency [AOR: 2.2 (1.3–3.6)] were significant independent risk factors associated with depression among older adults. Living with children [AOR: 0.33 (0.18–0.59)] and recreational activity [AOR: 0.54 (0.34–0.85)] were found to be significant protective factors against depression in older adults (Table 4).

**Table 4:** Logistic regression.

**Variable**	**Number of participants (n= 479)**	**Depression present (n=213)**	**Odds Ratio**	***P*- value**	**Adjusted Odds Ratio**	***P*-value**
**Age (in years)**						
Younger age group	350	150 (42.8%)	1.13 (0.56–1.7)	0.402		
Middle age group	95	48 (50%)	0.67 (0.22–0.89)	0.179		
Older age group	34	15 (44.11%)	Reference	Reference		
**Gender**						
Men	212	124 (58.4%)	1.02 (0.96–1.08)	0.09		
Women	267	89 (33.3%)	Reference	Reference		
**Religion**						
Hindu	462	207 (44.8%)	1.48 (0.78- 2.4)	1.2		
Muslim	17	6 (35.2%)	Reference			
**Marital status**						
Unmarried/divorced/separated	162	126 (77.7%)	Reference			
Married and living with spouse	317	87 (27.4%)	0.52 (0.48–0.57)	0.004		
**Education**						
Illiterate	201	97 (48.2%)	Reference			
Read only	82	40 (48.7%)	0.81 (0.33–1.94)	0.654		
Read and write	41	21 (51.2%)	0.44 (0.19–0.99)	0.049		
Primary	59	27 (45.7%)	0.35 (0.14–0.85)	0.021		
Middle	48	15 (31.2%)	0.39 (018–0.84).	0.017		
High school and above	48	13 (27.0%)	0.39 (0.19–0.79)	0.009		
**Employment**						
Unemployed	251	120 (47.8%)	Reference			
Unskilled	112	52 (46.4%)	0.94 (0.56–1.32)	0.75		
Semi-skilled	9	3 (33.3%)	0.54 (0.05–1.03)	0.27		
Skilled	40	12 (30%)	0.46 (0.36–0.56)	0.03		
Semi-professional	25	14 (56%)	1.38 (0.48–2.28)	0.34		
Professional	42	12 (28.5%)	0.43 (0.08–0.51)	0.02		
**Type of Family**						
Joint family (>5 members)	285	116 (40.7%)	Reference			
Nuclear (< 5 members)	194	97 (50%)	1.47 (1.2–1.6)	0.04		
**Poverty line (n**= **473, 6 non-response)**						
APL	395	163 (41.2%)	Reference			
BPL	78	45 (57.6%)	3.66 (2.8–4.4)	0.008		
**House**						
No house	59	33 (55.9%)	Reference			
Kucha	111	49 (44.1%)	0.50 (0.28–0.91)	0.39		
Mixed	125	59 (47.2%)	0.81 (0.5- 1.31)	0.15		
Pucca	184	72 (39.1%)	0.50 (0.45–1.13)	0.02		
**SES (Uday Parik)**						
0- lower class	255	129 (50.5%)	0.48 (0.44- 5.4)	0.56		
1- upper lower class	196	80 (40.8%)	0.73 (0.06–8.13)	0.78		
2- lower middle class	25	3 (12%)	3.66 (0.25–53.87)	0.37		
3- upper middle class	3	1 (33.3%)	Reference			
**Insurance**						
Yes	246	96	Reference			
No	233	117	0.78 (0.55- 1.12)	0.18		
**Substance abuse among the participants**						
No	366	117 (31.9%)	Reference			
Yes	113	96 (84.9%)	1.87 (0.9 – 2.4)	0.87		
**Physical activity**						
No	208	105 (50.4%)	Reference			
Yes	271	108 (39.8%)	0.58 (0.23–0.93)	0.004		
**Recreational activity**						
No	223	122 (54.7%)	Reference			
Yes	256	91 (35.5%)	0.47 (2.3–56)	0.001	0.542 (0.342- 0.859)	0.009
**Social Participation**						
Yes	158	63 (39.8%)	Reference			
No	321	150 (46.7%)	1.32 (0.89- 1.5)	0.15		
**Substance use in family members (current)**						
No	333	117 (35.1%)	Reference			
Yes	146	96 (65.7%)	15.1 (9.08–27.4)	< 0.021	16.7 (9.1–30.9)	0.001
**Financial Dependence**						
No	209	80 (38.2%)	Reference		2.2 (1.3–3.6)	< 0.001
Yes	270	173 (64.07%)	1.59 (1.08- 2.59)	0.01	Reference	-
**Physical dependence for self-care**						
No	471	207 (43.9%)	Reference			
Yes	8	6 (75%)	12.9 (2.9–56.2)	< 0.001	6.8 (1.3–35.4)	0.001
**Ever faced any elder abuse**						
No	385	145 (37.6%)	Reference			
Yes	94	68 (72.3%)	4.32 (2.6 – 7.9)	0.001	3.7 (2.1- 6.7)	0.001
**Living with their children/child**						
No	89	57 (64.04%)	Reference			
Yes	390	156 (40%)	2.679 (1.6–4.3)	< 0.001	0.33 (0.18–0.59)	0.023
**Adverse events in family**						
No	218	84 (38.5%)	Reference			
Yes	261	129 (49.4%)	1.77 (1.08- 2.2)	0.17	0.54 (0.34–0.85)	0.009
**Comorbidities**						
No	73	31 (42.4%)	Reference			
Yes	406	182 (44.8%)	1.01 (0.9- 1.4)	0.15		
**Non-communicable disease**						
No	82	34 (41.4%)	Reference			
Yes	397	179 (45.08%)	1.15 (0.71- 1.87)	0.54		
**Disability (Same person had multiple disabilities)**						
Locomotor	No	349	162	Reference			
	Yes	130	51 (39.2%)	0.74 (0.45–1.3)	0.27		
Hearing	No	458	467	Reference			
	Yes	21	12 (57.1%)	1.45 (0.95–1.94)	0.34		
Visual	No	437	457	Reference			
	Yes	42	22 (52.3%)	1.36 (0.35- 2.37)	0.23		

## Discussion

In the latest census of India, older adult men outnumbered older adult women in Odisha, but the distribution was similar to our study population in the younger age group (women: 51%, men: 49%) (17). The study participants were 55.8% women and 44.2% men, and the majority were in the younger age group (75%). Our study identified the prevalence of depression among older adults residing in rural Odisha as 44.4% after screening using GDS-15. Further grading with HDRS showed that among the depressed, 74.2% of the participants had mild depression and 1.9% had severe depression. Pilani et al. (5) conducted a meta-analysis to study the prevalence of depression among older adults in India from 1997 to 2016. The pooled prevalence of depression from 56 studies was 34.4% (29.3–39.6); however, a subgroup analysis from 28 studies in rural areas showed a prevalence similar to our study, 37.8% (29.9–45.9). In the same study by Pilani et al., a subgroup analysis from six studies in Eastern India showed a significantly higher prevalence of depression among older adults [47.9% (30.1–66.1), *P*-value: 0.0075] (5). The difference may be due to regional and geographic differences, including cultural background, social participation level, and healthcare access (18, 19). A study by Ashe et al. in the urban areas of Odisha also found a prevalence of depression of 44.2% (20). In our literature search, we could not find any studies to quantify depression among the rural older adults of Odisha. Thus, our investigation will be one of the first studies to assess the depression status of older adults in rural Odisha.

The prevalence of depression varies according to the scale used. A subgroup analysis of studies that used the GDS scale in the metanalysis by Pilani et al. (5) showed a pooled prevalence of 37.9% (31.5–44.5) (5). Another meta-analysis by Brooke Levis et al. found that screening tools such as GDS have a higher pooled prevalence (31%) than diagnostic tools (17%) (21). We also used HDRS to grade the severity and validate the diagnosis of depression in older adults screened positive by the GDS. The studies that used HDRS have shown a lower prevalence than our study. The survey among community-dwelling older adults in rural Haryana found the prevalence of depression to be 14.3% (22). A similar study in Himachal Pradesh found the prevalence of depression among older adults to be only 9.5% (23). A subgroup analysis of studies using HDRS in metanalysis by Pilania et al. (5) showed a pooled prevalence of depression among older adults of only 10.2% (5). The screening tools will have a higher prevalence than diagnostic tools. The difference is due to a higher sensitivity of screening tools (24). Positive screening by a screening tool should be confirmed by a psychiatrist for clinical diagnosis. However, screening followed by clinical diagnosis, treatment, and follow-up is the ideal management of depression in primary care settings (25).

In the present study, substance abuse among family members was the most vital associated factor [OR: 16.75 (9.082–30.926)] for depression among older adults residing in rural Odisha. A study by Ariyasinghe et al. (26) on women with spouses with substance use disorders in the rural community of Sri Lanka identified a significantly high prevalence (33.33%; CI: 25.93–40.73%) of major depressive disorders (26). The effects of children's substance use on parents are significantly less studied. Our literature search could not find any studies assessing the impact of children's substance use on older adults. This area should be studied more in the future to understand how the substance use of family members affects the mental health of older adults.

The present study could not find any significant association between depression and comorbidities, while physical dependency for self-care 12.9 (2.9–56.2) was significantly associated with depression. Even though several studies have found a significant association of depression with the morbidity status of older adults, our study suggests that morbidities without activity restriction and physical dependency are not risk factors for depression (27–32). In the book Physical Illness and Depression in Older Adults, Shaffer et al. (16) also studies that activity restriction can lead to depression in older adults (16). Schaffner explains that late-life changes like activity restriction following a physical illness can influence depression through the psychosocial pathway, which focuses on losing independence and control over one's life (33). A study among cancer patients found that activity restriction explained the significant additional variance in depression beyond the illness severity (34). A study by Lee et al. (15) found that after adjusting for general health and the severity of the morbidity, higher activity restriction was associated with higher depression. A cross-sectional study from rural Tamil Nadu also showed that being physically dependent (O*R* = 1.01; CI = 0.88–1.15) significantly affected depression among older adults (15). A study conducted in Sweden revealed that there was no discernible distinction in terms of illness or medication between older adults in primary care who were depressed and those who were not. However, it was noted that depressed older adults exhibited a significant correlation with the restriction of activities. Still, limiting activities had a significant association with depressed older adults (35). Further studies focusing on the activity restriction aspect of morbidities should be explored to associate the causation.

As per the psychosocial model of physical illness and the learned helplessness theory, losing control over one's life has a permanent casual attribute to to depressive disorders in older adults. The present study also found another related variable, i.e., financial dependency, as a risk factor for depression among older adults. A US study that followed up on the older adults for 4 years found that older adults who were economically disadvantaged had a risk of persistent depression (36). The exposure to financial instability plus an unstable and unsafe environment due to economic dependency increased the risk of depression in the older adults of the US study (36). The Financial Survey Status of Older People in India found that good financial quality ensured good health in older adults (37). Financial dependence also complicates the treatment of depression in older adults with low income (38). A study among older adults in rural Andhra Pradesh assessing economic dependency and its relation to depression found that 68% of the economically dependent were depressed (39). The study from Jaipur also saw that 48.3% of the financially dependent were significantly associated with depression (40). Economic dependency further reduces control over one's life, leading to depression in older adults.

In the present study, recreational activity was a significant protective factor [OR: 0.542 (0.342–0.859)]. In a 14-year follow-up study of community-dwelling older adults, recreational activity attenuated the association between depression and multiple morbidities [adjusted relative risk = 0.99, 95% confidence interval (0.98, 0.99); p = 0.001] (41). A Brazilian study assessing different domains of physical activity found that leisure and transport physical activity had a protective effect on depression. In contrast, physical work or household physical activity was a risk for depression in adults (42). Recreational activity assessed in our study included non-physical activities such as religious involvement, reading newspapers, listening to music, and watching TV. Physical activity was found to be significant in the univariate analysis [OR: 0.586 (0.234–0.938); *P*-value: 0.004] but was found to have no significance after multivariable analysis [0.897 (0.598- 1.196); *P*-value: 0.12]. This suggests that recreational activities without physical activity are more effective in the older adult population in reducing the risk of depression. However, our study was cross-sectional, and the temporality of the association could not be proved. The protective effects of recreational activities among older adults against depression should be explored further.

Older adult abuse is a neglected global health priority (43). A meta-analysis from 28 geographically diverse countries estimated a global pooled prevalence of 15·7% (95% CI 12.8–19.3) (44). The meta-analysis also found that psychological abuse was the most common, accounting for 11.2% of older adults (44). Older adult abuse is widely studied in developed countries. The close association between abuse and depression is also well-established. The most common abuse experienced by older adults is psychological and economic abuse, as evident from the meta-analysis from 28 countries (44). A bidirectional longitudinal Japan Gerontological Evaluation Study showed that people who experienced abuse had a 2.28 times higher risk of depression, and a follow-up after 3 years found those who were mildly to severely depressed before had a 2.23 times higher chance of experiencing abuse later on (45). Our study found elder abuse to be a significant risk factor for depression [AOR: 3.780 (2.113–6.760)]. A similar survey in Assam also found a positive correlation between abuse and depression (*r* = 0.619, *P* = 0.01) among older adults (46) A study from Nepal also found in a multivariable analysis that neglect (AO*R* = 2.995; CI: 1.249–7.181) and financial abuse (AO*R* = 4.728, CI: 1.836–12.173) contribute significantly to depression. Our study did not explicitly assess economic abuse, but financial dependency was significantly associated with geriatric depression.

The living status of older adults is another crucial risk factor identified in our study. Living with at least one child [OR: 0.374 (0.208–0.674); *P*-value: < 0.001] and living with a spouse [OR: 0.529 (0.485–0.573); *P*-value: 0.004] were found to be protective in univariate analysis. However, in multivariable analysis, only living with at least one child was significant [AOR: 0.333 (0.186–0.597); *P*-value: 0.025]. A cross-sectional study from rural Tamil Nadu also found that living with children (O*R* = 0.86, 95% CI = 0.77–0.97) protects older adults from depression (15). Other studies from different countries had varied results. A study from the migrant Russian population in the USA identified that older adults living alone only had depression while living with spouses or children were relatively protected from depression. Another study from China showed significant protection from depressive disorders only when the older adults lived with a spouse (*r* = 0.141), and the older adults living with children were more depressed (*r* = 0.189) (47). In Spain, not living with children was significantly associated with more depressive symptoms [unstandardized coefficient: 3.5 (0.9)], while living with children reduced depressive symptoms [unstandardized coefficient: 1.5 (0.8)] (48). The difference seen in different countries can be due to the cultural variations in each place. The SHARE study in Europe found that in areas where three-generation families are prevalent, the presence or contact with children is more important than that of a spouse or partner for the mental health of older adults (49). Our study was conducted in rural Odisha, where 37% were from three-generation families. The prevalent culture of rural Odisha consists of three-generation families, where the older adults live with children and grandchildren.

## Conclusion and recommendations

The study identified depression among 44% of older adults in rural Odisha, which needs to be addressed in the National Program for Health Care of the Elderly (NPHCE) through primary care settings. Quality of family life and independence were major factors affecting depression among older adults of rural Odisha. While substance use among family members and elder abuse was found to increase the risk of depression by 16 times and 3 times, respectively, and living with offspring decreased the risk of depression among older adults by 67%. After adjusting for comorbidities, physical activity, and adverse life events, physical dependency for self-care and financial dependency were found to be independent risk factors. Participating in recreational activities reduces depression among older adults by 45%. Strengthening NPHCE through community-based programs to improve the independence of older adults in family life should be prioritized. Furthermore, the mental health of older adults should be addressed from the primary healthcare setting onward, through screening, diagnosis, and management. Moving forward, large multicentric national studies identifying the mental health issues of older adults and studies identifying the temporality of associated factors should be considered.

## Data availability statement

The raw data supporting the conclusions of this article will be made available by the authors, without undue reservation.

## Ethics statement

The studies involving human participants were reviewed and approved by the Institute Ethics Committee, AIIMS Bhubaneswar. The patients/participants provided their written informed consent to participate in this study.

## Author contributions

AA: conceptualized the study with expert guidance and supervision from SPP and PB. AA: wrote the protocol, conducted the study, conducted the statistical analysis and drafted the initial protocol with inputs from SPP, PB, and SKP. All authors participated in interpreting the data and reviewed successive drafts of the manuscript for the intellectual content, read, and approved the final manuscript.
